# Supplementary material for: Identification of Differentially Expressed Genes and Molecular Pathways Involved in Osteoclastogenesis Using RNA-seq
Source: Genes (Basel). 2023 Apr 14;14(4):916. doi: 10.3390/genes14040916 (PMC10137460; doi:10.3390/genes14040916)

Figure S1a: Cluster analysis of upregulated differentially expressed genes.

Cluster 1

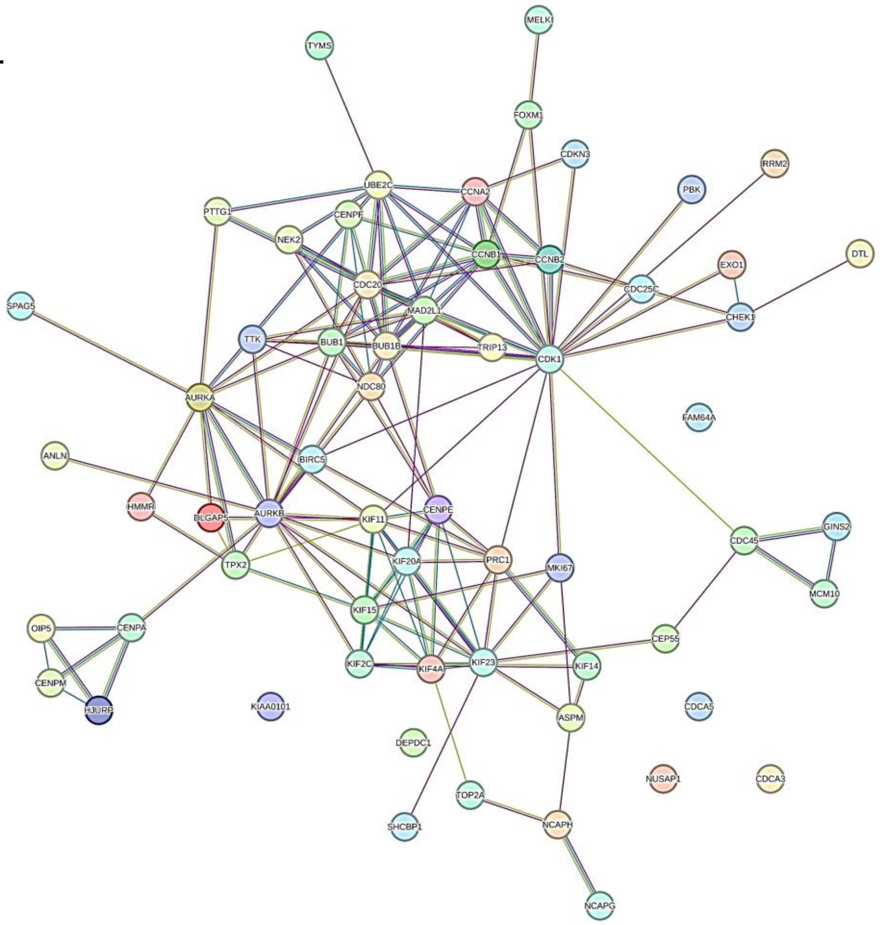

Cluster 2

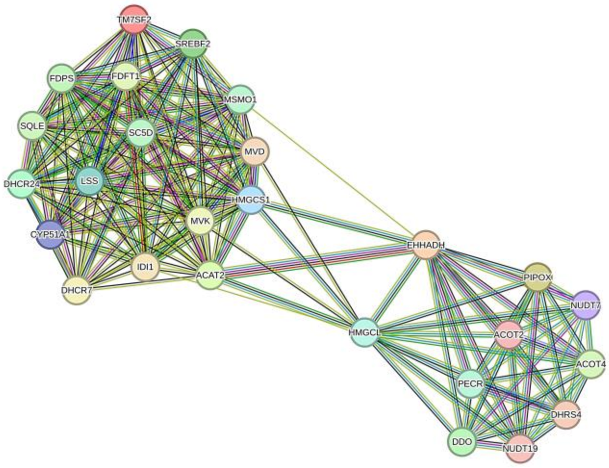

Cluster 3

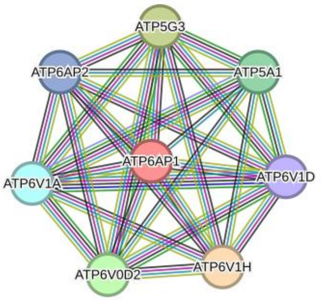

Supplement: Supplementary file 1 [file genes-14-00916-s001.zip › Figure S1a.pdf]
