# Supplementary material for: Identification of Differentially Expressed Genes and Molecular Pathways Involved in Osteoclastogenesis Using RNA-seq
Source: Genes (Basel). 2023 Apr 14;14(4):916. doi: 10.3390/genes14040916 (PMC10137460; doi:10.3390/genes14040916)
Supplement: Supplementary file 1 [file genes-14-00916-s001.zip › Table S2.pdf]

Table S2: Top 10 enriched pathways identified in each GO annotation for the upregulated gene set.

Category	Enriched terms	Count	Fold enrichment	FDR
Biological Process	cholesterol biosynthetic process	14	5.3	0.001
	cell division	56	2.02	0.001
	cell migration	42	2.1	0.004
	G2/M transition of mitotic cell cycle	16	3.8	0.01
	cell adhesion	69	1.7	0.01
	positive regulation of MAPK cascade	28	2.4	0.02
	chromosome segregation	17	3.1	0.04
	regulation of cyclin-dependent protein serine/threonine kinase activity	14	3.6	0.04
	cellular response to UV-B	6	10.2	0.046
	lipid metabolic process	32	2.1	0.046
Molecular Function	oxidoreductase activity	50	2.9	2.00E-08
	protein binding	1031	1.09	2.00E-05
	microtubule binding	45	2.2	3.03E-04
	protein kinase binding	67	1.7	0.001
	integrin binding	30	2.4	0.002
	identical protein binding	174	1.3	0.003
	actin binding	48	1.9	0.005
	collagen binding	17	3.3	0.005
	calmodulin binding	33	2.1	0.01
	ATP-dependent microtubule motor activity	12	3.8	0.029
Cellular Component	extracellular exosome	247	1.5	6.67E-10
	plasma membrane	460	1.2	1.27E-07
	cell cortex	37	3.1	2.83E-07
	endoplasmic reticulum membrane	125	1.7	5.41E-07
	membrane	256	1.4	5.41E-07
	apical plasma membrane	60	2.2	6.23E-07
	cell surface	83	1.8	1.46E-05
	cytoplasm	473	1.2	1.47E-05
	lysosomal lumen	24	3.4	1.65E-05
	integral component of plasma membrane	156	1.4	1.79E-05
