# Supplementary material for: Identification of Differentially Expressed Genes and Molecular Pathways Involved in Osteoclastogenesis Using RNA-seq
Source: Genes (Basel). 2023 Apr 14;14(4):916. doi: 10.3390/genes14040916 (PMC10137460; doi:10.3390/genes14040916)
Supplement: Supplementary file 1 [file genes-14-00916-s001.zip › Table S3.pdf]

Table S3: Top 10 enriched pathways identified in each GO annotation for the downregulated gene set.

Category	Enriched Terms	Count	Fold enrichment	FDR
Biological Process	inflammatory response	76	3.60	8.42E-19
	immune response	80	3.19	6.94E-17
	chemokine-mediated signaling pathway	25	6.83	6.03E-11
	chemotaxis	31	4.82	8.28E-10
	positive regulation of transcription from RNA polymerase II promoter	119	1.92	4.69E-09
	signal transduction	120	1.89	7.54E-09
	positive regulation of cytosolic calcium ion concentration	33	4.07	1.02E-08
	cell surface receptor signaling pathway	47	2.99	2.6E-08
	neutrophil chemotaxis	22	5.19	2.93E-07
	cellular defense response	18	6.49	3.04E-07
Molecular Function	transmembrane signaling receptor activity	43	4.27	1.42E-12
	C-C chemokine receptor activity	14	11.38	9.42E-09
	C-C chemokine binding	14	10.90	1.3E-08
	transcription factor activity	69	2.33	2.77E-08
	carbohydrate binding	32	2.99	1.77E-05
	chemokine receptor activity	10	9.84	3.28E-05
	signaling receptor activity	33	2.72	7.25E-05
	integrin binding	26	3.02	1.86E-04
	RNA polymerase II core promoter proximal region sequence-specific DNA binding	101	1.57	7.39E-04
	C-X-C chemokine receptor activity	6	16.02	8.36E-04
Cellular Component	plasma membrane	420	1.61	2.4E-26
	integral component of plasma membrane	177	2.33	8.34E-25
	external side of plasma membrane	76	3.25	2E-17
	cell surface	79	2.38	1.93E-10
	extracellular region	178	1.60	1E-08
	receptor complex	38	3.33	1.39E-08
	extracellular space	157	1.57	5.39E-07
	integral component of membrane	359	1.27	3.98E-06
	specific granule lumen	14	4.32	9.08E-04
	extracellular matrix	30	2.24	0.004
