# Supplementary material for: Identification of Differentially Expressed Genes and Molecular Pathways Involved in Osteoclastogenesis Using RNA-seq
Source: Genes (Basel). 2023 Apr 14;14(4):916. doi: 10.3390/genes14040916 (PMC10137460; doi:10.3390/genes14040916)
Supplement: Supplementary file 1 [file genes-14-00916-s001.zip › Table S4.pdf]

Table S4: GSEA KEGG pathway analysis for up and downregulated genes

Category	Enriched Pathways	Size	ES	NES	Nominal P-value	Q-value
Upregulated Pathways	KEGG Oxidative phosphorylation	89	0.66	2.16	0	0.001
	KEGG Valine Leucine and Isoleucine degradation	41	0.66	1.88	0	0.008
	KEGG Butanoate metabolism	25	0.73	1.87	0	0.007
	KEGG Propanoate metabolism	30	0.69	1.85	0	0.008
	KEGG Biosynthesis of unsaturated fatty acids	18	0.77	1.84	0	0.008
	KEGG Steroid biosynthesis	15	0.79	1.81	0	0.011
	KEGG Pyruvate metabolism	35	0.65	1.8	0.003	0.01
	KEGG Lysosome	118	0.51	1.76	0	0.016
	KEGG Citrate cycle TCA	27	0.66	1.75	0.002	0.016
	KEGG Gap junction	69	0.54	1.72	0	0.022
	KEGG Focal adhesion	166	0.48	1.69	0	0.023
	KEGG Glycolysis and gluconeogenesis	42	0.58	1.66	0.007	0.032
Downregulated Pathways	KEGG Cytokine cytokine interaction receptor	195	-0.51	-1.99	0	0.005
	KEGG Antigen processing and presentation	67	-0.58	-1.98	0	0.003
	KEGG Natural killer cell-mediated cytotoxicity	106	-0.54	-1.96	0	0.004
	KEGG Hematopoietic cell lineage	75	-0.56	-1.91	0	0.005
	KEGG Graft versus host disease	37	-0.62	-1.84	0	0.008
	KEGG Spliceosome	122	-0.48	-1.77	0	0.015
	KEGG N Nod-like receptor signaling pathway	56	-0.54	-1.74	0	0.018
	KEGG T-Cell receptor signaling pathway	101	-0.48	-1.72	0	0.02
	KEGG Basal cell carcinoma	39	-0.55	-1.7	0	0.023
	KEGG Primary immunodeficiency	32	-0.58	-1.69	0.002	0.022
