# Supplementary material for: Identification of Differentially Expressed Genes and Molecular Pathways Involved in Osteoclastogenesis Using RNA-seq
Source: Genes (Basel). 2023 Apr 14;14(4):916. doi: 10.3390/genes14040916 (PMC10137460; doi:10.3390/genes14040916)
Supplement: Supplementary file 1 [file genes-14-00916-s001.zip › Table S5a.pdf]

Table S5a: KEGG analysis of significantly upregulated genes identified in cluster analysis

Clusters	Pathways	Count	FDR
Cluster 1	Cell cycle	13	4.10E-15
	Oocyte meiosis	9	2.32E-08
	Progesterone-mediated oocyte maturation	8	8.73E-08
	Human T-cell leukemia virus 1 infection	7	2.10E-04
	p53 signaling pathway	5	2.96E-04
	Cellular senescence	6	3.29E-04
	Human immunodeficiency virus 1 infection	5	0.012
Cluster 2	Steroid biosynthesis	9	3.98E-15
	Metabolic pathways	22	6.85E-12
	Peroxisome	9	3.38E-10
	Terpenoid backbone biosynthesis	6	1.91E-08
	Butanoate metabolism	4	2.95E-04
	Valine, leucine and isoleucine degradation	4	0.001
	Lysine degradation	3	0.04
Cluster 3	Oxidative phosphorylation	7	5.15E-09
	Rheumatoid arthritis	5	3.40E-06
	Phagosome	5	2.31E-05
	Synaptic vesicle cycle	4	6.66E-05
	Metabolic pathways	7	4.16E-04
