# Supplementary material for: Identification of Differentially Expressed Genes and Molecular Pathways Involved in Osteoclastogenesis Using RNA-seq
Source: Genes (Basel). 2023 Apr 14;14(4):916. doi: 10.3390/genes14040916 (PMC10137460; doi:10.3390/genes14040916)
Supplement: Supplementary file 1 [file genes-14-00916-s001.zip › Table S5b.pdf]

Table S5b:KEGG analysis of significantly downregulated genes in cluster analysis

Clusters	pathways	Count	FDR
Cluster 1	Cytokine-cytokine receptor interaction	20	2.46E-24
	Viral protein interaction with cytokine and cytokine receptor	16	9.89E-24
	Chemokine signaling pathway	14	1.4E-15
	IL-17 signaling pathway	7	1.26E-06
	Amoebiasis	5	0.001
	NF-kappa B signaling pathway	5	0.001
	TNF signaling pathway	5	0.001
	Human cytomegalovirus infection	6	0.001
	Malaria	4	0.001
	Legionellosis	4	0.002
Cluster 2	Cytokine-cytokine receptor interaction	11	2.56E-07
	Inflammatory bowel disease	7	7.5E-07
	IL-17 signaling pathway	7	4.05E-06
	Hematopoietic cell lineage	7	4.05E-06
	Viral protein interaction with cytokine and cytokine receptor	7	4.05E-06
	Arrhythmogenic right ventricular cardiomyopathy	6	2.49E-05
	Cell adhesion molecules	7	3.03E-05
	ECM-receptor interaction	6	3.6E-05
	Hypertrophic cardiomyopathy	6	3.6E-05
	Rheumatoid arthritis	6	3.81E-05
